# Comprehensive Analysis of Affymetrix Exon Arrays Using BioConductor

**DOI:** 10.1371/journal.pcbi.0040006

**Published:** 2008-02-29

**Authors:** Michał J Okoniewski, Crispin J Miller

**Affiliations:** Whitehead Institute, United States of America

## Introduction

### 

#### Why BioConductor?

BioConductor [1] is a collection of open source software packages designed to support the analysis of biological data. BioConductor is written using the programming language R, which itself provides access to a wide range of tools for statistical analysis, data presentation, and visualization. BioConductor has more than 200 packages representing not only analytical tools but also data and annotation. It has a highly active international developers' community. For those unfamiliar with R and BioConductor, numerous books and tutorials exist (see, for example, [2–4]), and there are also very active e-mail mailing lists for both.

#### Why Exon Arrays?

Recent studies have shown that alternative splicing is prevalent—approximately 74% of all human multi-exon genes are predicted to be alternatively spliced (see Box 1 for an overview of the terminology), corresponding to about 50% of all human genes [5,6]. Alternative splicing participates in many pathways and processes; a detailed understanding of the cell must therefore include knowledge of the roles played by alternatively spliced genes and their products. Disruptions to the machinery of alternative splicing have also been implicated in many diseases, including neuropathological conditions such as Alzheimer disease, cystic fibrosis, those involving growth and developmental defects, and many human cancers [7,8]. A detailed understanding of disease and disease progression must therefore also involve an appreciation of the effects of changes in a cell's splicing behaviour.

#### From Genes to Exons.

Until recently, most microarrays considered transcription at the level of individual genes. They were, for the majority of genes, unable to distinguish between different isoforms, and, depending on the location of their probes, there was also the potential to miss certain transcripts entirely. Some groups have designed arrays to investigate genes by using many probes placed along their length in order to interrogate each exon separately. However, the number of features required to do this systematically, for the entire human genome, was prohibitively large.

**Figure 1:**
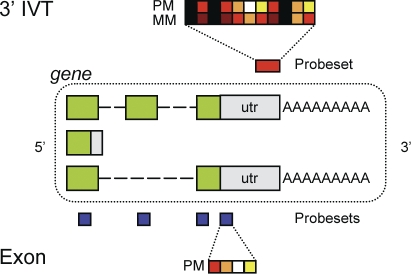
Differences in Array Design On standard 3′IVT, arrays such as the HGU133plus2 chip, each gene is typically targeted by a single probeset placed at the 3′ end of the transcript. These probesets consist of 11 Perfect Match spots and 11 paired Mis-Match spots in which the middle residue has been changed. Exon arrays have probesets placed against each exon along the length of the gene. Exon array probesets have no paired Mis-Match spots and four probes per probeset.

Advances in array technology have made it possible to design chips with increasingly smaller feature sizes. Affymetrix Exon arrays, for example, use more than 6.5 million features: the previous generation of Gene-level arrays had approximately 600,000. By removing the MM probes and reducing the number of probes within a probeset from 11 to 4, the total probeset count has been increased to ∼1.4 million, allowing probesets to be systematically placed along the full length of each gene (see Box 2 for an overview of the terminology; for more details on the design of the Affymetrix platform see the “Learning Center” on Affymetrix' Web site [9] or one of the many review articles (e.g., [10])). The aim has been to comprehensively target every known and predicted exon in the human genome (Figure 1).

An important point to appreciate, particularly as feature densities increase and arrays cover more and more of the transcribed genome, is that microarrays do not actually measure gene expression at all. Rather, they measure the abundance of RNA fragments in solution; gene expression is then subsequently inferred from the data.

#### How Reliable Are the Data?

Exon arrays are very different from the previous generation of (3′IVT) arrays, such as the HGU133plus2 chip. Many changes have been made, including the removal of the MM probes, a reduction in the number of PM probes in each probeset from 11 to 4, changes in array design, and changes to the protocols used for RNA preparation. Given the large number of changes, it is important to assess the performance of the arrays. In [11] and [12] (available at [9]), this was done by comparing them to HGU133plus2 arrays, themselves extensively validated. Exon arrays were found to produce data of similar quality to that from the earlier arrays. A more detailed discussion of the differences between exon and 3′IVT arrays, including approaches to Quality Control, can be found in Text S1.

## A General Workflow for Exon Array Analysis

**Figure 2:**
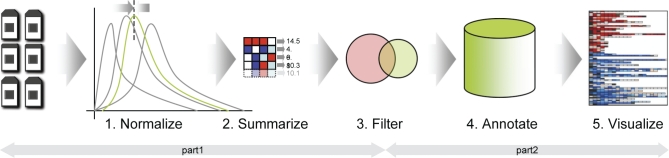
Exon Arrays Can Be Analysed Using Standard Approaches Developed for 3′IVT Arrays A standard pipeline for array processing involves 1. normalizing the arrays, 2. generating expression summaries, 3. filtering on correlation, fold-change, and/or statistical significance to select interesting probesets, 4. mapping those probesets to their target transcripts by annotation, and 5. visualization and downstream analysis.

The community has converged on a relatively standard set of approaches for analysing existing 3′IVT arrays (Figure 2).

A similar approach can be applied to exon array data. In particular, the same algorithms for analysing 3′IVT arrays can be used for Exon chips up until step 3. Novel strategies must be employed in the last two steps because of the need for more complex annotation to deal with the richness of the data produced by the arrays.

## Pre-Processing Exon Array Data

### 

#### Step 1: Normalization.

Biological and technical variations inject enough variability into the system for it to be inappropriate to directly compare raw data for each individual sample without first pre-processing (or “normalizing”) the data in order to bring them together. Many techniques exist, most working on the assumption that on average, the majority of data points between samples are unchanged. Thus, a straightforward procedure might simply scale each array to the same mean intensity, perhaps after removing outliers. A more invasive approach might also require each array to have the same standard deviation, or to have the same-shaped distributions. Normalization can be performed on the raw feature/spot levels, or on the probeset data after expression summary. Most recent algorithms perform normalization first, and the same techniques that were applied to 3′IVT arrays are applicable to exon arrays (see the supplement worked examples, Text S2).

#### Step 2: Expression summary.

Expression summary is the process by which the values for each individual probe in a probeset are summarised to generate a single value for that probeset. Again, many techniques exist, but all perform some kind of weighted average, possibly with background correction.

#### RMA.

Observations about the relative utility of MM probes led to the design of RMA [13]. Importantly, RMA doesn't use MM probes, making it directly applicable to exon arrays.

After normalization (by default, quantile), the data are fitted to a global model of expression and probe affinities. For each probeset, PM_ij_ = *e_i_* + *a_j_* + *ɛ_ij_*, where *e* is a chip effect and *a* represents the probe affinity for the *j^th^* probe on the *i^th^* array.

The model is based on the hypothesis that the intensity measured for a perfect match spot is dependent on three things: the amount of material available to bind to the spot (the chip effect, *e*), the “stickiness” of the probe (the probe affinity, *a*), and a measurement error (*ɛ*). RMA works by estimating, for each probeset, values for the probe and chip effects that would result in the pattern of PM values observed in the data. This process of “model fitting” is performed using an algorithm called median polish, a fast numeric technique for estimating model parameters.

#### PLIER.

PLIER, proposed by Affymetrix [14], is a similar algorithm to RMA. It also fits a global model, but starts from a slightly different set of assumptions. In theory, PLIER can use MM probes; its behaviour without MM is qualitatively similar to RMA. PLIER offers some additional parameters that may be used to tune the model.

#### GeneBASE.

An alternative, model-based approach designed specifically for exon array summarization was proposed recently in [15].

#### Step 2½: Filtering to remove poorly performing probesets—Detection calls.

While expression summary algorithms generate an estimated value for the abundance of transcript in solution, they do not provide a measure of how reliable that figure is. Exon arrays, which do not have paired MM probesets (and cannot use the approach of [16]), have a separate pool of 25,000 background probes designed not to match exactly to the transcriptome. A detection above background (DABG) score can be calculated for each probeset by matching PM probes to members of the background pool with the same GC content and measuring the relative distance between the two [17]. In [11], DABG calls were found to be useful in removing poor performing probesets prior to analysis.

#### Step 3: Identifying significant probesets and dealing with multiple testing.

Statistical tests (e.g., a *t*-test) are used to assess how likely changes in differential expression are to have occurred by chance. With a single test, a *p*-score of, say, 0.05, might be acceptable; there is a 5% chance of chasing shadows. Microarrays present a challenge, because they perform thousands (or in the case of exon arrays ∼1.4 million) tests in parallel. At standard thresholds, many probesets will be identified by chance (approximately 5% of the array: ∼70,000). Multiple testing correction aims to address this by choosing more stringent thresholds in order to reduce the false positive rate. This must be balanced against the incorrect rejection of real results—i.e., the false negative rate.

A popular strategy is to try to find a threshold such that the set of probesets that pass the test is expected to contain a specified number of false positives—i.e., the false discovery rate (FDR). FDR is appealing not least because it is easy to interpret and provides a useful measure of the reliability of the dataset in question.

Exon arrays present further challenges, because of the size of the datasets and because the non-independence between probesets makes it difficult to accurately compute the FDR [18]. That said, current techniques can still be applied to exon array data, and do yield sizable numbers of differentially expressed probesets.

## Mapping to Annotation

A number of strategies have been developed that re-annotate array data by in silico searches against the genome and/or transcriptome. A popular approach is to identify transcripts (or genes) targeted by multiple probesets. These are then combined to produce larger consensus probesets [19,20]. One advantage of this approach is that it reduces the total number of probesets involved in the analysis, and thus has a positive effect on the multiple testing issues discussed above.

Care must, however, be taken when applying these approaches to exon arrays, because the amount of evidence supporting each probeset is varying, and some probesets have been placed on the array with much less confidence than others. Strategies that aim to define new probesets using annotation must deal with these. Another issue is that model-based algorithms such as PLIER and RMA make the assumption that all probes within a probeset target the same thing. When alternate splicing is involved (particularly given the potential for novel and/or overlapping transcripts), this assumption may not hold.

An alternate strategy is to use the standard probeset definitions provided by the manufacturer, and to apply annotation to the data only after the “interesting” probesets have been selected. This is the approach taken here. Annotation can also, in some circumstances, be used successfully to pre-filter the data before statistical analysis by, for example, removing all probesets except those that match known exons in the Ensembl genome database. Again, this can have a beneficial effect on the FDR.

## X:MAP, a Database of Exon Array Annotation

X:MAP is a database of exon array annotations built by performing an in silico search between exon array probes and the entire genome. These data are stored in a relational database and associated with a copy of Ensembl [21]. X:MAP has a Web-based genome browser (based on Google Maps) that allows the relationship between probesets, the genome, and gene structure to be browsed [22], and an associated BioConductor package, *exonmap*, which communicates with X:MAP and provides access to its data from within R. The design and implementation of the database is described in more detail in [23].

*Exonmap* supports mappings backward and forward between potential probe hits, probesets, exons, transcripts, and genes. The database allows these comparisons to be performed for Ensembl genes, ESTs, and Genscan predictions.

Filtering is provided to allow probesets to be selected or excluded, based on whether they map to introns, exons, transcripts, or genes. It is also important to consider the hit-specificity of different probes within a probeset, since a substantial proportion (about 6%) of probes match identically to more than one Ensembl exon. Additional functions are provided to filter based on whether probesets contain “Multiply Targeted” probes that match the genome in more than one location. This is important because a small but substantial number of probesets are capable of hybridizing to the genome at multiple locations, and because much of the genome is predicted to be transcribed. Mapping of probesets to the genome is described in more detail in [23].

Exonmap provides some basic functions to load expression data into R (based on the “affy” package). In order to preprocess the array data, it is necessary for the software to know which features on the surface of the array correspond to which probesets. This is generally specified using a “Chip Definition File,” or CDF. Custom CDF data for exon Human, Mouse, and Rat arrays can be found in the “downloads” section of X:MAP [24].

Once data are loaded they can be processed using standard analysis tools, such as *limma*, or *siggenes*, in order to identify sets of interesting probesets. A simple utility function *pc()* is provided to perform pairwise comparisons between two replicate groups and to generate fold-changes and unadjusted *p*-values. We will use it here for convenience—more sophisticated analyses are of course possible, and generally advisable—however, since the focus here is on the annotation tools, a simple strategy is sufficient to generate an initial probeset list for further analysis.

The data on which this tutorial was generated is part of a comparison between MCF7 and MCF10A cell lines. It can be downloaded from [25].

### 

#### Hello world.

The simplest route to a list of differentially expressed probesets (the exon array equivalent of “ world”) is as follows:

**1** > library(exonmap)

**2** > raw.data <- read.exon()

**3** > raw.data@cdfName <- “exon.pmcdf”

**4** > x.rma <- rma(raw.data)

**5** > pc.rma <- pc(x.rma,“group”,c(“a”,”b”))

**6** > keep <- (abs(fc(pc.rma)) > 1) & tt(pc.rma)< 1e-4

**7** > sigs <- featureNames(x.rma)[keep]

Line 1 simply loads the package.

Line 2 loads the expression data into the *ExpressionSet* object *raw.data*, and relies on the presence of a file (by default called ‘covdesc') that defines the names of the .cel files to be loaded and their associated experimental parameters. The covdesc file used here is as follows:

group

ex1MCF7_r1.CELa

ex1MCF7_r2.CELa

ex1MCF7_r3.CELa

ex2MCF10A_r1.CELb

ex2MCF10A_r2.CELb

ex2MCF10A_r3.CELb

When *read.exon()* loads the data, the additional experimental parameters defined in *covdesc* are loaded into the *ExpressionSet*, and can be retrieved by the function *pData()*.

Line 3 takes a little more explanation. The raw array data is stored in a .CEL file, which records, for each of the ∼6 million features on the array, the raw unprocessed spot intensity. These must be grouped into probesets, as specified by a Chip Definition File (CDF). Line 3 tells BioConductor which CDF file defines the array layout by setting the name of the .cdf package (“exon.pmcdf”). CDF files can be downloaded from [24].

Line 4 uses RMA to normalize and generate expression summaries for the probesets. To use PLIER, the following code can be substituted:

**8** x.pli <- justPlier(raw.data, usemm=F, normalize=T, norm.type=”pmonly”, concpenalty=0.08)

The concpenalty parameter can be used to adjust how PLIER deals with probesets that have very different values on a small proportion of the arrays in a project (i.e., outlier probesets). We have found it can be useful to set it to a higher value than the default (0.000001) when processing small numbers of arrays. Otherwise, PLIER can tend to see genuine differences between arrays as noise. This is translated into reduced fold changes for these probesets. PLIER's tuning parameters are described in more detail in [26].

Line 5 uses *pc()* to calculate fold-changes and *t*-test *p*-values for each probeset on the array. Note that it is using the data loaded in from the *covdesc* file to specify that the comparison should be between samples labelled “a” and “b” in the column “group”.

Line 6 uses the result of the pairwise comparison to find all probesets with an absolute fold-change greater than 1—all data are on a log2 scale, so at least 2-fold differentially expressed—with an unadjusted *p*-value less than 10^−4^, and line 7 fishes the names of these probesets out from the *raw.data* object. Clearly, more sophisticated approaches (using, for example false discovery rates to set thresholds) can, and probably should, be substituted here.

## Mapping to Annotation

Irrespective of how the probeset list is identified, these must then be mapped to the genome and to gene annotations. The aim of exonmap is to provide methods to answer questions such as these:

1. Which probesets hit exons, introns, transcripts, and/or genes?

2. Which probesets hit between known genes—and do they match ESTs or Genscan predictions?

3. Which genes/transcripts are differentially expressed or alternatively spliced?

The basic approach is to provide a series of functions *X.to.Y()* that allow lists of identifiers to be mapped between the different levels of annotation. First, however, it is necessary to connect to the database:

**9** > xmapDatabase(“Human”)

This relies on a configuration file specifying how to connect to the database. Full details are in the package vignette and installation instructions.

**10** > probeset.to.exon(sigs[1:5],list.out=TRUE)

$‵2318663‵

[1] “ENSE00000738016”

$‵2318675‵

[1] “ENSE00000737793”

$‵2318682‵

[1] “ENSE00001435048” “ENSE00000737789”

$‵2318693‵

[1] “ENSE00000401699”

Note that probeset ‘2318682' matches to two different exons (a quick search on X:MAP shows that these are from two different transcripts from the same gene—PER3). Other parameters can force the results to be returned as a vector, to be filtered to remove duplicates (see the manual page of [27]—i.e., *?probeset.to.gene* for more details). Similar functions can be used to perform mappings backward and forward between exons and genes (*exon.to.gene*, *gene.to.exon*), transcripts, etc. Functions also exist to get detailed information for exons, transcripts and genes—for example:

**11** > transcript.details("ENST00000377541")

transcript_id stable_id seq_region_id name

ENST00000377541 226810 ENST00000377541 226034 1

seq_region_start seq_region_end seq_region_strand

ENST00000377541 7766967 7786605 1

biotype status description external_db_id

ENST00000377541 protein_coding KNOWN NA 2000

db_display_name display_label

ENST00000377541 UniProtKB/TrEMBL Q8TAR6_HUMAN

description

ENST00000377541 PER3 protein (Period homolog 3) (Drosophila).

### 

#### Filtering based on probeset location.

At line 7, an initial probeset list was generated. Many of these probesets match outside Ensembl genes, whilst others match inside genes, but outside exons. Filtering functions are provided to select or exclude these probesets from the probeset list, as follows:

**12** > sigs.exonic <- select.probewise(sigs, filter="exonic")

Intronic and intergenic probesets can be selected in a similar way, and the analogous function *exclude.probewise()* allows probesets to be filtered out of a list.

Some probesets contain probes that match the genome at multiple sites. These can be selected or removed as follows:

**13** sigs.mt <- select.probewise(sigs, filter="multitarget")

Filtering criteria are based on the number of times each probe within a probeset matches the genome, a gene, or an exon. The aim is to identify those “well-behaving” probesets that consist of probes that match only once to the feature of interest.

## Searching for Alternative Splicing—Splicing Index and Splicing ANOVA

Exon arrays enable genome-wide, high-throughput searches for alternative splicing events [28]. There are many intuitive methods of assessing if the gene is alternatively spliced (e.g., the coefficient of variance of the exonic probesets within a gene).

A simple, but effective method, the “splicing index”, was proposed in [29]. Each exonic probeset is normalized by dividing its expression by the gene-level summary value for the entire gene to yield a normalized intensity (NI). The splicing index is then calculated simply as a “fold change” between the two normalized levels:

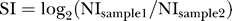



The method depends on the selection of the gene-level expression summary [14]. In exonmap, the mean (or median) value for all exons is used [19].

**14** > si.sig <- splicing.index(x.rma, sig.gene, "group", c("a","b"))

Gene level summaries are calculated, by default, as the median expression of ‘well-behaving' exon probes (where ‘well-behaving' means not multiply-targeted, and hitting an exon with all four probes in the probeset).

Searches for alternative splicing may also be done for experiments with multiple treatments. In this case, in place of a simple index, there is an ANOVA model, tested for each probeset. The null hypothesis is that there is no alternative splicing for the gene, and the NI level for each probeset should therefore not be significantly different across the samples. *p*-Values are calculated for all the probeset-treatment pairs; alternative splicing events are selected as those with low *p*-values:

**15** #in an experiment with more than two replicate groups

**16** > splanv <- splanova(x.rma, my.genes,"group","a",thr=0.05)

**17** > fval(splanv)

## Downstream Analysis—Visualization Tools

**Figure 3:**
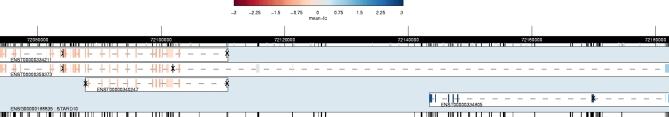
Expression for STARD10, Mapped to Known Isoforms and Coloured According to Fold Change between MCF7 and MCF10A

A set of visualization tools is available within *exonmap*. *plot.gene()*, for example, will plot the structure of the specified gene, coloured according to the expression data (Figure 3). Minimum, maximum, mean, and median intensity are all possible, as are mean and median fold-changes between sample groups. By default, the gene's average expression is calculated and used to colour the overall gene; transcripts and exons are coloured relative to that. The default colouring is thus very similar in philosophy to the splicing index.

*gene.graph()* plots expression data as a line plot, with exons coloured and placed according to location, on the *x*-axis of the graph. By default, intronic and multiply targeted probesets are not shown; parameters allow this to be changed.

**Figure 4:**
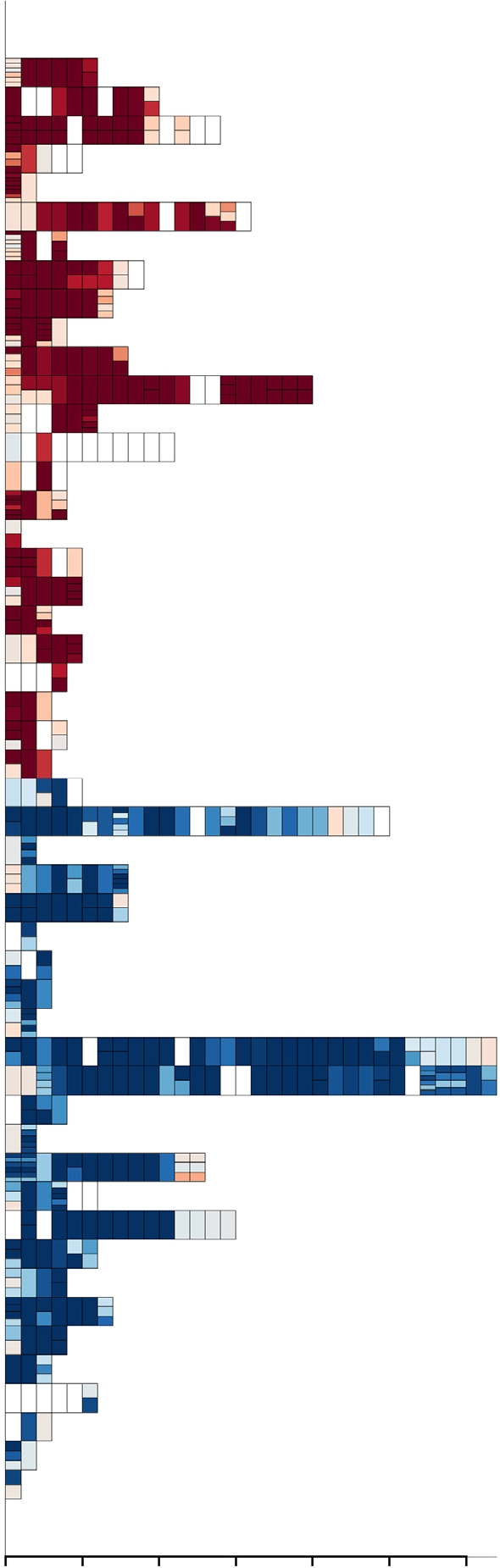
20 Differentially Expressed Genes Selected for High Variance within Their Probesets Each row corresponds to a gene, each rectangle to an exon. Exons are arranged in sequence order. If an exon is targeted by multiple probesets, these are stacked vertically within that exon. The plot is coloured by fold change between MCF7 and MCF10A (red, up in MCF7; blue, up in MCF10A).

Finally, *gene.strip* produces a heatmap-style representation for a list of genes. Each rectangle in the plot corresponds to an exon, and is coloured by expression, as before. By default, introns are not drawn; when they are, the *x*-axis represents actual sequence position, exons are drawn as (by default) black rectangles, and probesets by larger rectangles coloured by gene expression (Figure 4).

## Finally

We hope you found this tutorial useful. Further examples and workflows can be found in the package vignettes and in Text S1 and Text S2 including the pursuit of novel exons and transcription from intergenic regions.

## Supporting Information

Text S1 Difference between Exon and Previous Generation Affymetrix Arrays (52 KB PDF)

Text S2 Example Workflow (93 KB PDF)

**Box 1. Alternative Splicing Terminology**

A **gene** is a region of the genome that is transcribed into RNA. A transcribed RNA molecule is referred to as a **transcript**. The RNA produced by genes that encode protein sequences is called **messenger RNA** or** mRNA**. In eukaryotes, genes can contain **exons** and **introns**. The introns are removed from the initial transcribed RNA (or **pre-RNA**) by **splicing**. Splicing can also be used to remove exons. By selectively retaining different sets of exons within the spliced transcript, cells can produce multiple **isoforms** from a single gene, and, if subsequently translated, multiple proteins. This process is known as **alternative splicing**.

**Box 2. Design of Affymetrix Arrays**

Affymetrix expression arrays use a set of **features** (often referred to as “**spots**”) designed to recognize each molecule of interest. Each feature consists of millions of identical single-stranded 25-mer nucleotide **probes**, each designed to hybridize to a specific transcript. On a gene-level array, such as the HGU133plus2 chip [30], each of these **Perfect Match (PM)** features is accompanied by an adjacent **Mis-Match (MM)** feature in which the middle residue is changed. Hybridization conditions are designed to maximise binding to the PM features while minimizing binding to the MM ones. Each MM feature can therefore be used to provide a measure of probe specific background for its PM partner. Multiple PM/MM pairs are used for each transcript. On most gene-level arrays, 11 PM/MM pairs are used per transcript, and the complete set of 22 features is referred to as a **probeset**.
