# Supplementary material for: Comprehensive Analysis of Affymetrix Exon Arrays Using BioConductor
Source: PLoS Comput Biol. 2008 Feb 29;4(2):e6. doi: 10.1371/journal.pcbi.0040006 (PMC2323405; doi:10.1371/journal.pcbi.0040006)
Supplement: Supplementary file 1 [file pcbi.0040006.sd001.pdf]

Differences between 3'IVT and Exon arrays

Design of Exon arrays

A variety of annotation sources have been used to design Exon arrays, including not only well characterized genes, but also gene predictions with varying degrees of confidence and with alternate predictions of start and stop sites. These include RefSeq, GenBank, Ensembl, and prediction from tools such as Genscan [1].

Changes in protocol

In order to process a sample, RNA must be extracted and then prepared for hybridization to the chip. This includes the incorporation of a fluorescent label and at least one round of amplification in order to yield enough material. The standard protocol for existing arrays does this using reverse transcription, primed from the poly-A tail that is found at the 3' end of each mRNA sequence. (This is why these arrays are often referred to as 3' IVT arrays.)

There is, therefore, the potential for the 3' end of each transcript to be over-represented in the final hybridization cocktail, and, if the initial RNA is fragmented or degraded, for the reverse transcription process to perform less well, reducing representation at the 5' end. For these reasons, the majority of probesets on 3' IVT arrays are designed to target the 3' end of the gene. Another consequence of placing probesets in the 3' un-translated region (the **3'UTR**) of the transcript is that they will not detect changes further upstream. Work with exon arrays shows that this is a relatively frequent event.

Exon arrays, which have probesets across the full length of the gene, require a different strategy. Priming is instead performed using a set of random hexamers, allowing reverse transcription to be initiated at multiple locations across the transcript [2]. Description of protocols can be found at the Affymetrix website [3], and full details of the protocols used by the Cancer Research UK Affymetrix service can be found at [4].

Detection calling

For 3'IVT arrays, a detection calling algorithm can be used. It assesses how far the PM signals for a given probeset are above the accompanying MM values

With the previous generation, 3' IVT arrays, probesets were designed against the 3' end of each transcript, and there was a general 1-1 relationship between probeset and transcript. In some circumstances, such as when there are alternate poly-Adenylation signals, multiple probesets were placed against each gene, resulting in a one-many mapping [5]. Certain probes can also hybridize to more than one transcript, possibly from different genes, adding further complexity.

References and further reading

1. Affymetrix (2005) Exon Probeset Annotations and Transcript Cluster Groupings. Affymetrix Whitepaper.

2. Affymetrix (2007) Whole Transcript (WT) Sense Target Labeling Assay Performance.

3. Affymetrix. http://www.affymetrix.com

4. Paterson Institute GeneChip Microarray Service. http://bioinformatics.picr.man.ac.uk/mbcf/index.jsp

5. Okoniewski MJ, Miller CJ (2006) Hybridization interactions between probesets in short oligo microarrays lead to spurious correlations. BMC Bioinformatics 7: 276.
