# Supplementary material for: Comprehensive Analysis of Affymetrix Exon Arrays Using BioConductor
Source: PLoS Comput Biol. 2008 Feb 29;4(2):e6. doi: 10.1371/journal.pcbi.0040006 (PMC2323405; doi:10.1371/journal.pcbi.0040006)
Supplement: Supplementary file 2 [file pcbi.0040006.sd002.pdf]

A simple workflow for analysing Affymetrix exon arrays in BioConductor

Crispin J. Miller and Michał J. Okoniewski
cmiller@picr.man.ac.uk

Preamble

The aim of this workflow is to provide examples of how the BioConductor package *exonmap* can be used to analyse Affymetrix exon array data. It exploits where possible existing tools and strategies for analysing 3'IVT arrays (such as the HGU133plus2 array). This includes standard approaches for normalization and pre-processing, including RMA, GCRMA and PLIER, and standard methods for filtering for differential expression, including LIMMA, SAM and simple fold-change and t-test based approaches.

Please note that this script is intended to work with versions **1.4.0** and above of exonmap.

We assume a basic knowledge of R and BioConductor.

Overview

The basic strategy is shown in Figure 1. Expression data are loaded, normalized and pre-processed in order to identify a set of 'interesting' genes. These might be selected according to fold-change or correlation or selected on the basis of statistical significance, as is common with existing 3'IVT arrays. Given a set of 'interesting' probesets, annotation is then used to map these probesets to the genome, and thus to the features they target. Here we consider a simple pairwise comparison between two cell lines, MCF7 and MCF10A.

Step 1: loading the data

Exonmap uses the core affy packages within BioConductor to represent array data. In order to do this, a non-standard .cdf package is used; this can be downloaded from http://xmap.picr.man.ac.uk.

Thus, to load exon array data, and to specify the name of the cdf package to use, the following three lines of code are sufficient:

```
library(exonmap)

raw.data <- read.exon()

raw.data@cdfName <- "exon.pmcdf"
```

`read.exon()` relies on a text file (called, by default, `'covdesc'`) containing data such as this:

```
                  group
ex1MCF7_r1.CEL    a
ex1MCF7_r2.CEL    a
```

```
ex1MCF7_r3.CEL    a
ex2MCF10A_r1.CEL  b
ex2MCF10A_r2.CEL  b
ex2MCF10A_r3.CEL  b
```

in order to specify which CEL files to read, and to supply additional annotation data describing the samples.

Step 2: normalization and expression summary

Pre-processing is done in the usual way, but with the proviso that there are no paired mis-match spots on the arrays, so algorithms such as mas5 won't work, and PLIER needs to be told not to use the mis-match spots:

```
x.rma <- rma(raw.data)

#or plier can be used:

x.pli <- justPlier(raw.data, usemm=F, normalize=T,
norm.type="pmonly", concpenalty=0.08)
```

Step 3: identifying differentially expressed probesets

Exonmap has a couple of simple functions to calculate fold-changes (and unadjusted t-test p-scores). `pc()` can be used to perform a pairwise comparison between two sets of arrays. `fc()` and `tt()` can be used to extract the fold-changes and p-scores produced for each probeset:

```
pc.rma <- pc(x.rma,"group",c("a","b"))

keep <- (abs(fc(pc.rma)) > 1) & tt(pc.rma)< 1e-4

sigs   <- featureNames(x.rma)[keep]
```

Similarly, other popular tools can be used, such as `limma`:

```
library(limma)

design <-
as.matrix(cbind(MCF7=c(1,1,1,0,0,0),MCF10A=c(0,0,0,1,1,1)))

fit <- lmFit(x.rma,design)

cont.matrix <- makeContrasts(MCF7vsMCF10A=MCF7-
MCF10A,levels=design)

fit2 <- contrasts.fit(fit,cont.matrix)

fit2 <- eBayes(fit2)


result <- decideTests(fit2,lfc=1)

keep   <- result@".Data" != 0
```

```
20 limma.sigs   <- rownames(result@".Data")[keep]
```

The result is a list of significant probesets.

Step 4: mapping to annotation

First we must connect to the X:MAP database which will provide the annotation:

```
21 xmapDatabase(“Human”)
```

Now, given a list of ‘interesting’ probesets (such as those identified in line 9, on the basis of fold-change and p-score), we can select just those probesets that hit exons:

```
22 exonic <- select.probewise(sigs,”exonic”)
```

A small but significant proportion of probesets on the array contain probes that might hybridize in more than one location. These can be filtered out:

```
23 exonic.filtered <- exclude.probewise(exonic,”multitarget”)
```

At this stage we have a list of ‘interesting’ exon-targeting probesets that match the genome uniquely in one location. These can now be mapped to genes:

```
24 genes <- probeset.to.gene(exonic.filtered)
```

An obvious question becomes, for each of these genes, do all their probesets show the same fold-change? One way to evaluate this would be, for each gene, to calculate the variance of the fold change for its probesets. If the variance is low, then the probesets are consistent with one another, if the variance is high, then different probesets are doing different things between the samples. This provides a simple metric for distinguishing between differentially expressed and alternatively spliced genes:

```
25 splicevar <- function(a) {
26   r <- apply(a[,1:6],1,function(b) {
27     mean(b[1:3] - b[4:6])
28   })
29   m <- mean(r)
30   v <- var(r)
31   c(v,m)
32 }
```

Thus, `splicevar` is a function that takes a matrix of expression data, each row a probeset, each column, a sample. It assumes that the first three samples contain the MCF7 data, the last 3, the MCF10A data. It computes the mean fold change between the cell lines for each probeset, and places the results in *r*. The mean and variance of these values are then computed for the given set of probesets.

In a moment, we will use `splicevar` to calculate the variance (it also calculates the mean; we will use that later) for each of the genes we identified at line 24. First,

however, we need to find, for each of these genes, their exon-targeting probesets. Exonmap provides a utility function that does this, and maps them to their expression data:

```
gep <- gene.to.exon.probeset.expr(x.rma,genes)
```

It produces a data.frame:

```
gep[1:3,]
```

```
  ex1MCF7_r1.CEL ex1MCF7_r2.CEL ex1MCF7_r3.CEL ex2MCF10A_r1.CEL
1       6.375762       5.836923       6.433112         6.485873
2       6.084308       6.756105       6.689520         7.081683
3       6.168754       6.426774       6.219463         6.277609
  ex2MCF10A_r2.CEL ex2MCF10A_r3.CEL            gene            exon probeset_id
1         6.069215         6.028865 ENSG00000162572 ENSE00001066428         470
2         7.000371         6.738188 ENSG00000162572 ENSE00001066429         475
3         6.067723         6.196594 ENSG00000162572 ENSE00001066430         480
  probeset_name probe_count
1       2315713           4
2       2315718           4
3       2315723           4
```

We can split this on the gene name, to produce a list of data.frames, one for each gene:

```
l <- split(gep,gep$gene)
```

…and then apply our splicevar function to each entry in the list, using sapply:

```
vars <- sapply(l,splicevar)
```

We now have a table of means and variances, one for each of our genes. (Note that the table has two rows, the first, the variances, the second, the means, and *N* columns, one for each gene). They are distributed like this:

```
#vars

plot(density(vars[1,],na.rm=TRUE))

#means

plot(density(vars[2,],na.rm=FALSE))
```

We can then partition the data based on this:

```
DE <- vars[,(vars[1,] < 1)]

AS <- vars[,(vars[1,] > 2)]
```

Other approaches

Alternatively, we can compute the splicing index, or use an ANOVA based approach:

```
si.sig <- splicing.index(x.rma, genes, "group", c("a","b"))

#For experiments with more than two replicate groups

#splanv <- splanova(x.rma,
genes[1001:1010],"group","a",thr=0.05)
```

Visualization

We can generate global plots for all of our genes. For example, we can sort genes according to their variances, and by looking at the sign of their means, determine in which direction the majority of probesets are changing:

```
up <- vars[2,] > 0
dn <- vars[2,] < 0
o.up <- order(vars[1,up],decreasing=T)
o.dn <- order(vars[1,dn],decreasing=T)
#get the 10 most ‘up’ and ‘down’ genes
to.plot <- c(colnames(vars[,up][,o.up[1:10]]),colnames(vars[,dn][,o.dn[1:10]]))
gene.strip(to.plot,x.rma,list(1:3,4:6))
```

We can also plot data for individual genes:

```
plot.gene(genes[1],x.rma,list(1:3,4:6))
gene.graph(genes[1],x.rma,list(1:3,4:6),type="mean-int")
```

All three plotting functions have a variety of parameters to change how they plot their data – see the man pages for more details.

Novelty

Some probesets target genes, but fall between the well characterized exons that form the ENSEMBL genes. These can be found and filtered as before, using:

```
intronic <- select.probewise(sigs,”intronic”)
intronic.filtered <- exclude.probewise(intronic,”multitarget”)
```

Similarly, probesets that target between known genes can identified with:

```
intergenic <- select.probewise(sigs,”intergenic”)
intergenic.filtered <- exclude.probewise(sigs,”multitarget”)
```

These can then be mapped to Genscan and EST based transcript predictions (currently using the development version of the package, version 1.1.14 or above). For example:

```
xmapExtras()
genscan <- probeset.to.transcript(intronic,db=”prediction”)
EST     <- probeset.to.transcript(intronic,db=”est”)
```

Finally, the functions `xmapProbeset()`, `xmapGene()`, etc. will open up an instance of the X:MAP genome browser pointing to the location of the specified feature.
